# Redox‐Addressable Single‐Molecule Junctions Incorporating a Persistent Organic Radical**

**DOI:** 10.1002/anie.202116985

**Published:** 2022-04-05

**Authors:** Saman Naghibi, Sara Sangtarash, Varshini J. Kumar, Jian‐Zhong Wu, Martyna M. Judd, Xiaohang Qiao, Elena Gorenskaia, Simon J. Higgins, Nicholas Cox, Richard J. Nichols, Hatef Sadeghi, Paul J. Low, Andrea Vezzoli

**Affiliations:** ^1^ Department of Chemistry University of Liverpool Crown Street Liverpool L69 7ZD UK; ^2^ School of Engineering University of Warwick Coventry CV4 7AL UK; ^3^ School of Molecular Sciences University of Western Australia Crawley Western Australia 6009 Australia; ^4^ School of Chemistry South China Normal University Guangzhou 510006 P.R. China; ^5^ Research School of Chemistry Australian National University Canberra ATC 2601 Australia; ^6^ Stephenson Institute for Renewable Energy University of Liverpool Peach Street Liverpool L69 7ZF UK

**Keywords:** Molecular Devices, Molecular Electronics, Radicals

## Abstract

Integrating radical (open‐shell) species into non‐cryogenic nanodevices is key to unlocking the potential of molecular electronics. While many efforts have been devoted to this issue, in the absence of a chemical/electrochemical potential the open‐shell character is generally lost in contact with the metallic electrodes. Herein, single‐molecule devices incorporating a 6‐oxo‐verdazyl persistent radical have been fabricated using break‐junction techniques. The open‐shell character is retained at room temperature, and electrochemical gating permits in situ reduction to a closed‐shell anionic state in a single‐molecule transistor configuration. Furthermore, electronically driven rectification arises from bias‐dependent alignment of the open‐shell resonances. The integration of radical character, transistor‐like switching, and rectification in a single molecular component paves the way to further studies of the electronic, magnetic, and thermoelectric properties of open‐shell species.

## Graphical abstract

A verdazyl radical, stable in its open‐shell state at ambient conditions, has been used to fabricate single‐molecule devices that show enhanced charge‐transport efficiency, electrochemical transistor behaviour, and rectification. All the properties arise from the presence of an unpaired electron, strongly localised on the verdazyl core.

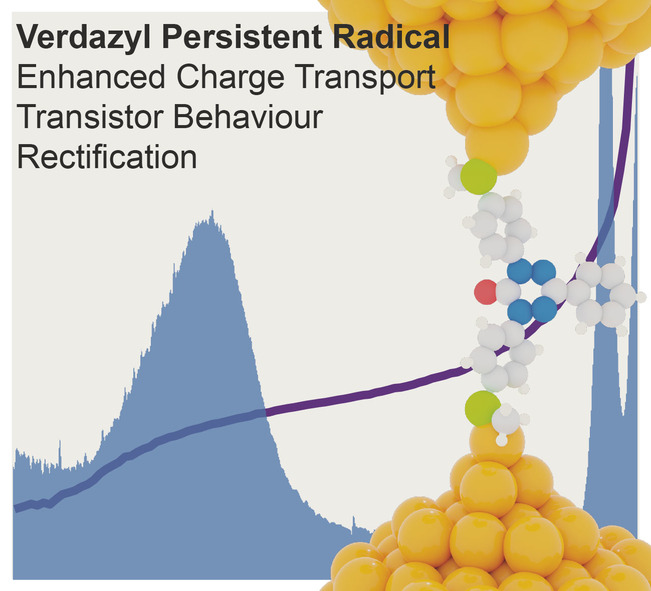

## Introduction

Molecular radicals feature open‐shell electronic structures (i.e. an odd number of electrons) and, as a consequence, they are often highly reactive and sensitive to atmospheric oxygen and moisture. Nevertheless, since Gomberg's successful synthesis of the triphenylmethyl (trityl) radical in 1900,^[1]^ several organic open‐shell compounds that are stable under ambient conditions have been prepared and characterised, and they have shown outstanding performance in electronic devices such as batteries, transistors and light‐emitting diodes.^[2]^ When incorporated into metal|molecule|metal junctions, the presence of singly‐occupied orbitals results in sharp transport resonances in the transmission function *T*(*E*) (the probability for an electron with given energy *E* to tunnel through the molecular wire) near to the electrode Fermi level.^[3]^ These resonances are highly sought‐after features in molecular electronics,[^4^, ^5^, ^6^] imparting desirable properties such as rectification or non‐linear behaviour.^[7]^ As the conductance *G* of a molecular junction can be estimated using the Landauer formula *G* = *G*_0_ *T*(*E_F_*) where *G*_0_ = 2*e* ^2^/*h* ≅ 77.48 μS, a high transmission coefficient (i.e. a resonance) at the Fermi level results in high charge transport efficiency. The high slope of *T(E)* vs *E* near the Fermi energy offered by radical systems is also predicted to give rise to enhanced Seebeck coefficients,^[8]^ leading to enticing prospects for the design of molecular thermoelectric materials,[^6^, ^9^, ^10^] whilst the presence of an unpaired electron provides the basis from which to extend electronics to spintronics.[^11^, ^12^] Despite the rich promise of radical‐based molecular electronics, attempts to date to incorporate persistent organic radicals into single‐molecule junctions at room temperature have resulted in loss of the open‐shell character,^[13]^ or in a modest enhancement of electrical conductance.[^8^, ^14^] The significant increase in charge transport efficiency predicted by theoretical studies^[6]^ has only been observed at cryogenic temperatures.[^15^, ^16^] These observations are not entirely surprising, as the open‐shell molecular components used in previous studies had either a highly delocalised radical state (e.g. polyhalogenated trityl compounds)[^17^, ^18^] or a low oxidation potential (e.g. Blatter radical, −0.29 V vs Fc/Fc^+^)^[13]^ which leads to electron transfer when in contact with the metallic electrodes, and hence quenching of the open‐shell state. To overcome these issues, methods involving the in situ generation of radical states have been successfully adopted, with electrochemical gating or chemical redox processes providing the required long‐term stability of the molecular radical within the junction.[^19^, ^20^, ^21^, ^22^, ^23^] The search for a single‐molecule electronic device based on a native, persistent radical species that is stable at room temperature and under ambient atmospheric conditions is therefore ongoing.

**Figure 1:**
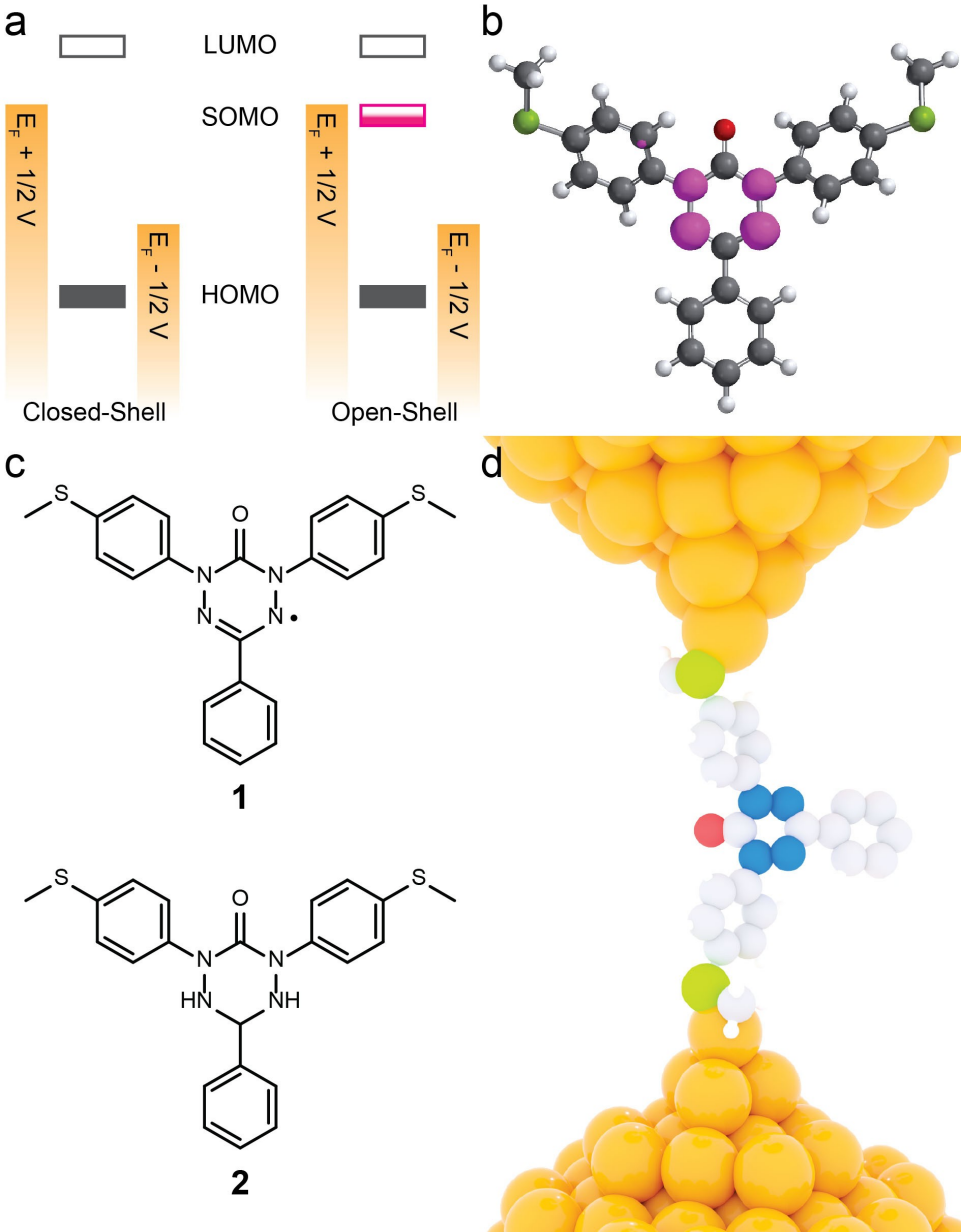
Rationale and proposed experiments. a) Example of the energy level diagram for closed‐shell and open‐shell material within a molecular junction. The SOMO (magenta) will sit in the HOMO–LUMO gap and is better aligned with the Fermi levels of the two metallic electrodes, resulting in higher conductance; b) Spin density map (magenta) for the oxoverdazyl radical **1**, with isosurfaces at 0.005 e^−^ Bohr^−3^ (B3LYP/6‐31G DFT, Spartan ‘18); c) Structures of the compounds used in this study; and d) cartoon depiction of **1** coordinated to two Au nanoelectrodes as a single‐molecule junction. Atom colours in (b, d): C=grey; H=white; O=red; S=green; N=blue; Au=orange.

In this letter, we demonstrate that the 6‐oxoverdazyl derivative **1** maintains its radical character when fabricated into single‐molecule junctions between two Au electrodes, showing significantly enhanced conductance when compared with its closed‐shell tetrazin‐3‐one precursor **2** (Figure 1). We attribute our finding to the localisation of the radical state on the nitrogen atoms of the oxoverdazyl core (Figure 1b),^[24]^ that allows **1** to maintain its open‐shell nature even when coordinated to the two metallic nanoelectrodes at room temperature. The presence of a radical state in the single‐molecule junction is confirmed by reversible in situ electrochemistry of **1** within the junction, which is exploited to engineer single‐molecule transistor behaviour. Furthermore, technologically relevant properties such as rectification and nonlinear current–voltage behaviour are imparted to the single‐molecule device by the presence of a strongly localised half‐filled molecular orbital.

## Results and Discussion

**Figure 2:**
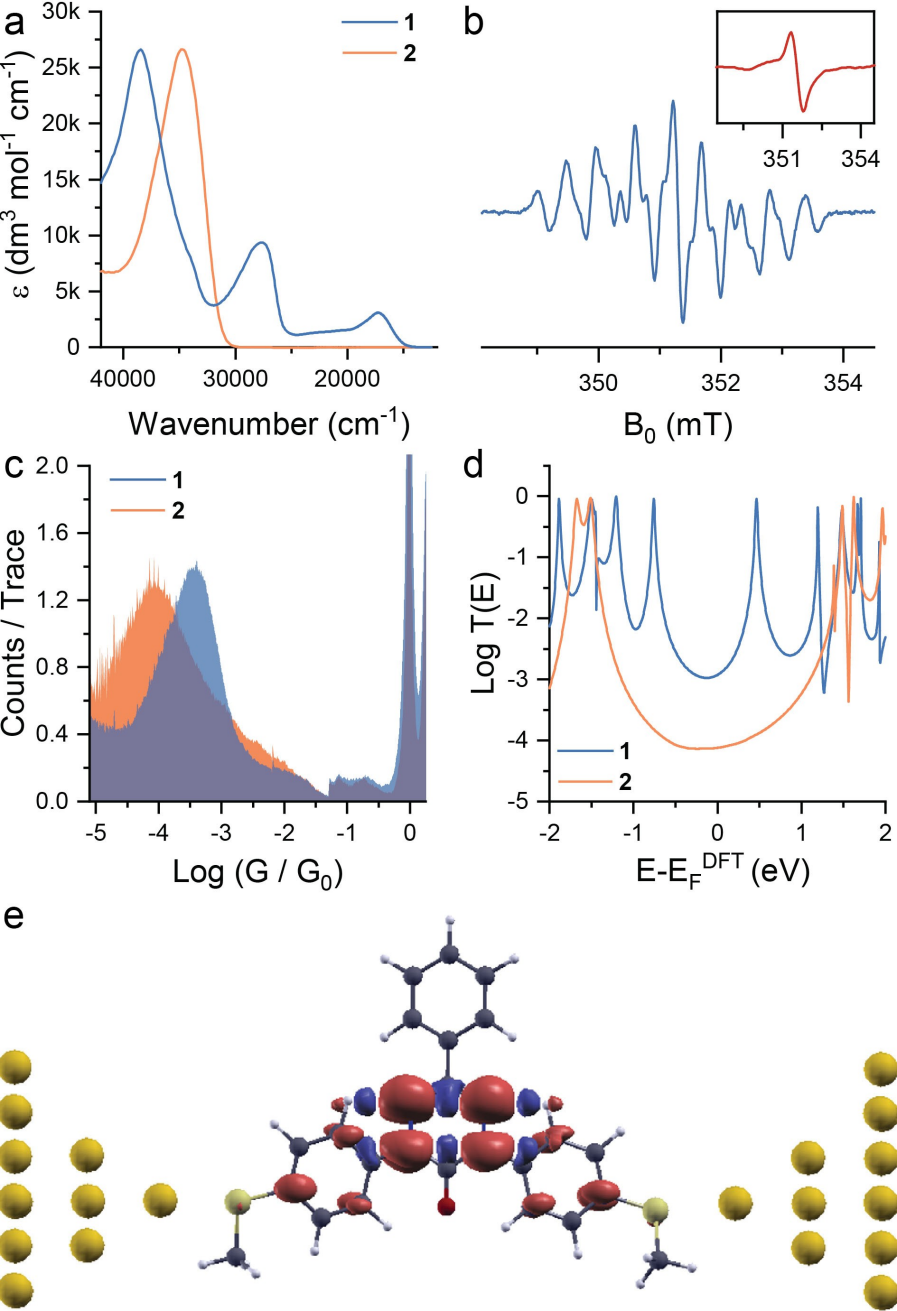
Experimental determination of open‐shell properties of **1**. a) UV/Vis spectra of **1** and **2** in CH_2_Cl_2_ solution; b) cw‐EPR (X‐band ≈9.4 GHz) of **1** in solution (1 mM in CH_2_Cl_2_, blue) and adsorbed on a Au substrate (inset, red); c) Single‐molecule conductance histogram for **1** and **2** measured in mesitylene (1 mM) at 200 mV bias (>5000 individual scans for each compound, with no data selection, compiled with 100 bins per decade, and normalised to the number of scans as counts/trace); d) Transmission curves ($\log\left(T\left(E\right)\right)vs\;E-E_{F}^{DFT}$ ) for **1** and **2** referenced to the Fermi level of the electrodes. e) DFT‐calculated spin density of **1** between Au nanoelectrodes. In the case of the 6‐oxoverdazyl radical **1**, the transmission function *T*(*E*) is the average transmission for both spin‐up and spin‐down contributions.

The synthesis and chemical characterisation of **1** and **2** are described elsewhere.^[25]^ Evidence of the persistent radical state of **1** is given by the presence of adsorption bands at 17 300 cm^−1^ (≈580 nm, corresponding to an optical band gap of 2.13 eV) and 27 600 cm^−1^ (≈360 nm, 3.44 eV), assigned to transitions involving singly‐occupied orbitals,[^25^, ^26^, ^27^] while **2** only shows an absorption band in the ultraviolet corresponding to a π–π* (HOMO–LUMO) transition (Figure 2a). In addition, continuous‐wave electron paramagnetic resonance (cw‐EPR) spectroscopy of **1** exhibited a line‐shape characteristic of 6‐oxoverdazyl derivatives (Figure 2b),[^24^, ^28^, ^29^, ^30^] with a hyperfine structure that can be rationalized in terms of the unpaired electron spin coupling to two sets of equivalent *I*=1 nuclei (two sets of equivalent ^14^N atoms), consistent with the calculated distribution of the spin density over these atoms. EPR measurements of **1** adsorbed on a Au substrate confirm the retention of its open‐shell character (Figure 2b, inset). Its spectrum has a simpler structure due to line broadening (from the loss of molecular tumbling) and increased delocalization of the unpaired spin (for further details see the Supporting Information).

Single‐molecule junctions of **1** and **2** were fabricated using the scanning tunnelling microscope—break junction (*STM‐BJ*) method in order to characterise their charge‐transport properties.^[31]^ In this technique, a Au STM tip is driven into a Au substrate under DC bias *V* until a metallic contact having conductance ≫*G*_0_ is formed. The tip is then withdrawn at a constant speed (10 nm s^−1^ in this study) in a dilute solution of the target molecular wire, while continuously monitoring the current *I* at high acquisition rate (>10 kS s^−1^) as a function of the relative tip‐substrate position *z*. During the withdrawal process, the metallic contact is continuously thinned until it is reduced to a point contact having conductance *G*_0_. As the withdrawal continues, the point contact will break, generating a nanogap of ≈0.5–0.6 nm due to snapback of the electrodes.^[32]^ Molecules from solution that have self‐assembled along the metallic filament can bridge the resulting nanogap, thus fabricating the single‐molecule junction.^[33]^ The withdrawal process is continued to stretch the molecule into its extended state and to finally rupture the molecular junction. The tip is then driven again into the substrate and the process is repeated several thousand times to acquire statistically significant data. Data are acquired in the form of *G*(*z*) traces (conductance *G* is calculated using Ohm's law *G* = *I*/*V*) that bear multiple plateaux at: (i) integer multiples of *G*_0_, characteristic of conductance quantisation of single‐channel conductors such as Au atoms; and (ii) at values ≪*G*_0_, characteristics of transport through partially open quantum channels such as molecular wires. Data are then compiled in semilogarithmic histograms and heatmaps for further statistical analysis. Plateaux in the *G*(*z*) traces result in peaks in the histograms and in high‐density areas in the heatmaps, yielding information on the most probable conductance values and molecular extension of the junction. Information about the instruments and materials^[34]^ used in this study and the data acquisition and analysis process^[35]^ can be found in the Supporting Information.

The 6‐oxoverdazyl radical **1** displayed efficient charge transport, with a conductance peak centred at 10^−3.4^ *G*_0_, almost one order of magnitude more conductive than its closed‐shell precursor **2** (10^−4.3^ *G*_0_) (experiments in mesitylene in Figure 2c; experiments in air available in the Supporting Information). Further analysis shows that in all cases the molecule is stretched to its extended state in the junction, as features are evident in the heatmaps (available in the Supporting Information) up to an electrode separation of ≈0.65 nm (≈1.25 nm accounting for the snapback^[36]^ of the electrodes) that is in good agreement with the molecular length calculated using density‐functional theory (DFT, Spartan'18, B3LYP/6‐31G, 1.35 nm for **1** and 1.29 nm for **2**). As compounds **1** and **2** have similar molecular geometry and share the same linkers to the electrodes (thioanisole), no significant difference in the structure of the junction is expected and differences in molecular conductance are therefore attributed to differences in the electronic structures of the heterocyclic molecular cores.

To better understand these results, quantum transport calculations were used to compute the zero‐bias transmission probability for electrons with energy *E* through junctions formed from **1** and **2**, details of which are given in the Supporting Information. A pair of sharp resonances in the transmission function *T*(*E*), lying close to the Fermi level, are calculated for the junction formed from **1**. These resonances arise from the singly‐occupied (SOMO, α‐HOMO, or spin‐up) and singly‐unoccupied (SUMO, β‐LUMO, or spin‐down) orbitals of the radical, as also demonstrated by local density of state calculations (see Supporting Information). Our calculations of **1** between gold electrodes (Figure 2e) shows that the spin density is localised on the central ring even when the molecule is in the junction, in a way similar to the gas phase calculations (Figure 1b). The overlap of these resonances raises *T*(*E*) for **1** above 10^−3^ *G*_0_ over a wide range of electron energies around the Fermi level. In contrast, the transport profile of the closed‐shell tetrazin‐3‐one **2** shows that the Fermi level lies towards the bottom of a dip in the transmission function, within a wide HOMO–LUMO gap (Figure 2d), leading to significantly lower molecular conductance.

**Figure 3:**
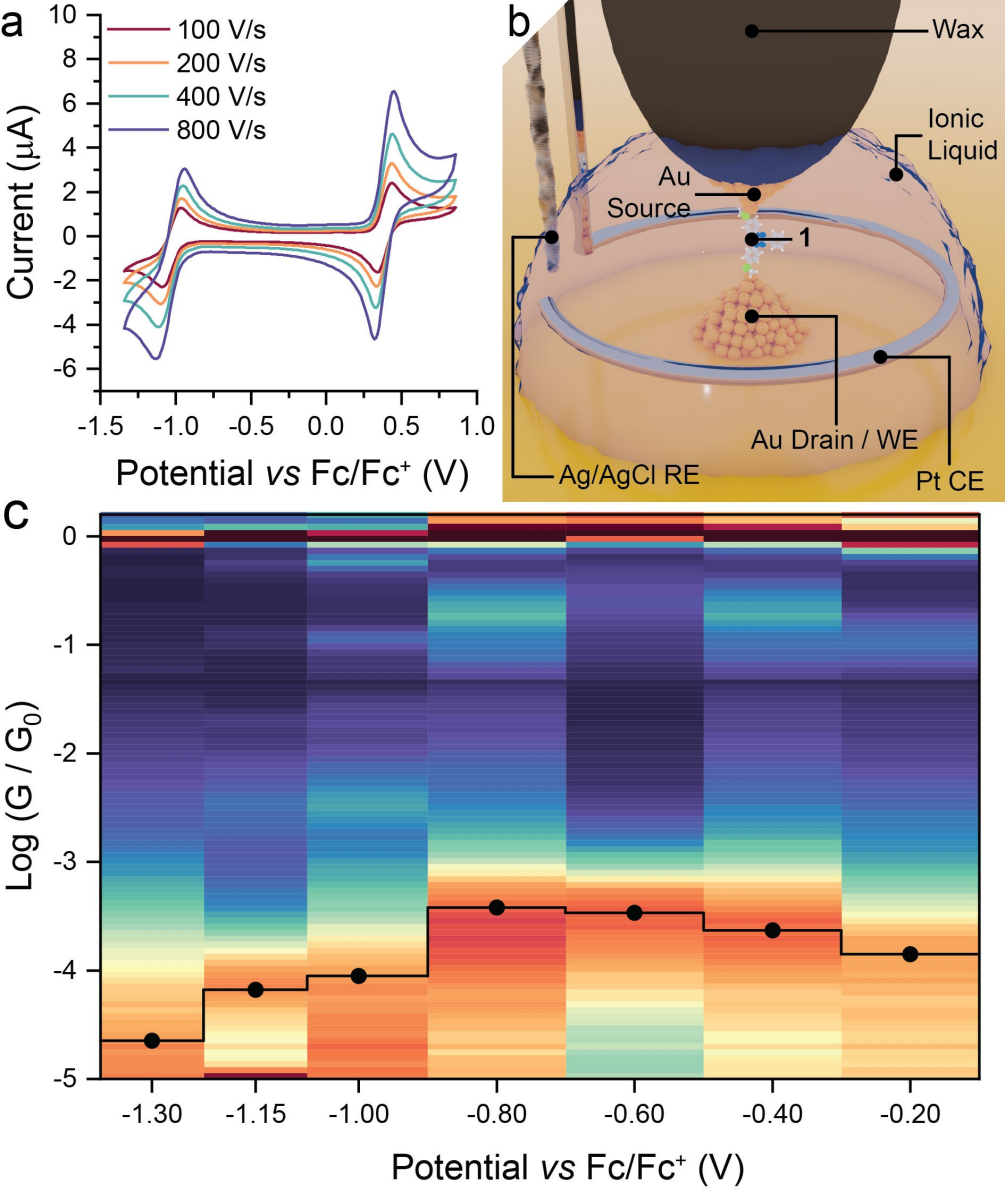
Electrochemical Experiments. a) Cyclic voltammetry of **1** in degassed CH_2_Cl_2_, with 0.1 M tetrabutylammonium hexafluorophosphate as supporting electrolyte. Potentials referenced to the ferrocene/ferrocenium redox couple using a decamethylferrocene standard. b) Depiction of the 4‐electrode setup used in the single‐molecule electrochemical gating studies with the molecular junction in place. c) Heatmap (blue=low counts; red=high counts) of electrochemically‐gated single‐molecule conductance data (compiled with 10 bins per decade from more than 5500 individual scans at each potential) across the electrochemical window explored in the ionic liquid 1‐butyl‐3‐methylimidazolium triflate, with points indicating the most probable conductance determined from a Gaussian fit of the conductance histogram at each potential overlaid in black. Potential referenced to the ferrocene/ferrocenium redox couple as internal standard.

As with other examples of 6‐oxo‐verdazyl radicals,^[29]^ compound **1** undergoes electrochemically reversible one‐electron oxidation (to the corresponding monocation) and one‐electron reduction (to the monoanion) processes (Figure 3a). Both the reduction and oxidation processes occur at relatively high positive (+0.36 V) or negative (−1.03 V) potentials, which confirm the excellent stability of the 6‐oxoverdazyl radical in the presence of metallic substrates.

To verify the involvement of the well‐aligned singly‐occupied orbital in the increased charge transport efficiency of **1**, the electrical characteristics of the STM‐BJ were further explored in an electrochemical environment, with a bipotentiostat maintaining a potential between the working electrode (the STM Au substrate and tip) and a Pt counter electrode *vs* an Ag/AgCl reference electrode. The STM tip was insulated with Apiezon wax^[37]^ to reduce the faradaic current and constantly biased against the substrate by the bipotentiostat to ensure the two Au electrodes could act as source and drain (Figure 3b).^[38]^ Experimental measurements were made in the ionic liquid 1‐butyl‐3‐methylimidazolium triflate,[^20^, ^35^, ^39^] and under these conditions the setup displayed an open circuit potential of approximately −0.4 V vs the ferrocene/ferrocenium (Fc/Fc^+^) redox couple. The conductance of **1** remained at values around those obtained without electrochemical control from −0.2 to −0.8 V vs Fc/Fc^+^. In this potential window, a small electrostatic gating effect can be observed, with conductance slowly increasing as the potential is made more negative (Figure 3c). As the potential is further decreased to −1 V vs Fc/Fc^+^, near to the reduction potential of **1**, the junction conductance abruptly drops by almost one order of magnitude and continues to decrease as the potential is further reduced. Importantly, the more conductive open‐shell state could be recovered by reversing the potential, highlighting the reversible transistor behaviour of junctions fabricated with **1** (see Supporting Information).

The initially observed electrostatic gating effect provides confirmation that **1** offers a resonance lying close to the Fermi level in the junction, with the abrupt decrease in junction conductance at more negative gate potentials indicating that this resonance is removed when **1** is reduced. In the potential window where **1** is in its open‐shell state (−0.2→−0.8 V vs Fc/Fc^+^), conductance increases as the potential is made more negative, indicating that the transport resonance due to the SUMO (or β‐LUMO) is initially at energy *higher* than the Fermi level of the electrodes. As the gate potential passes −1.0 V vs Fc/Fc^+^ and **1** is reduced, the slope of the conductance/potential dependence suddenly changes sign, and the transport resonance is now due to the HOMO of the reduced **1**, at energy *significantly lower* than the Fermi level. Conductance data for **1** in the full electrochemical window explored (−1.3→0.5 V vs Fc/Fc^+^) is provided in the SI. Attempts to obtain single‐molecule conductance data at potentials >0.5 V vs Fc/Fc^+^ (where **1** would be oxidised to the monocationic state) were not possible due to increased instrumental noise at these potentials. We hypothesise this is due to the insolubility of the cationic species in the ionic liquid used in this study, which therefore precipitates from solution preventing further junction fabrication. Changes in the properties of ionic liquids in high electric fields[^40^, ^41^] have been reported in the literature, with corresponding modifications in their ion solvation capabilities.[^42^, ^43^] To corroborate this hypothesis, we performed voltammetric studies of **1** in 1‐butyl‐3‐methylimidazolium triflate (available in the Supporting Information). In this environment, the reduction of **1** to the anionic state shows a quasi‐reversible behaviour, but its oxidation is completely irreversible, with only the forward wave **1→1^+^** appearing during cyclic voltammetry.

**Figure 4:**
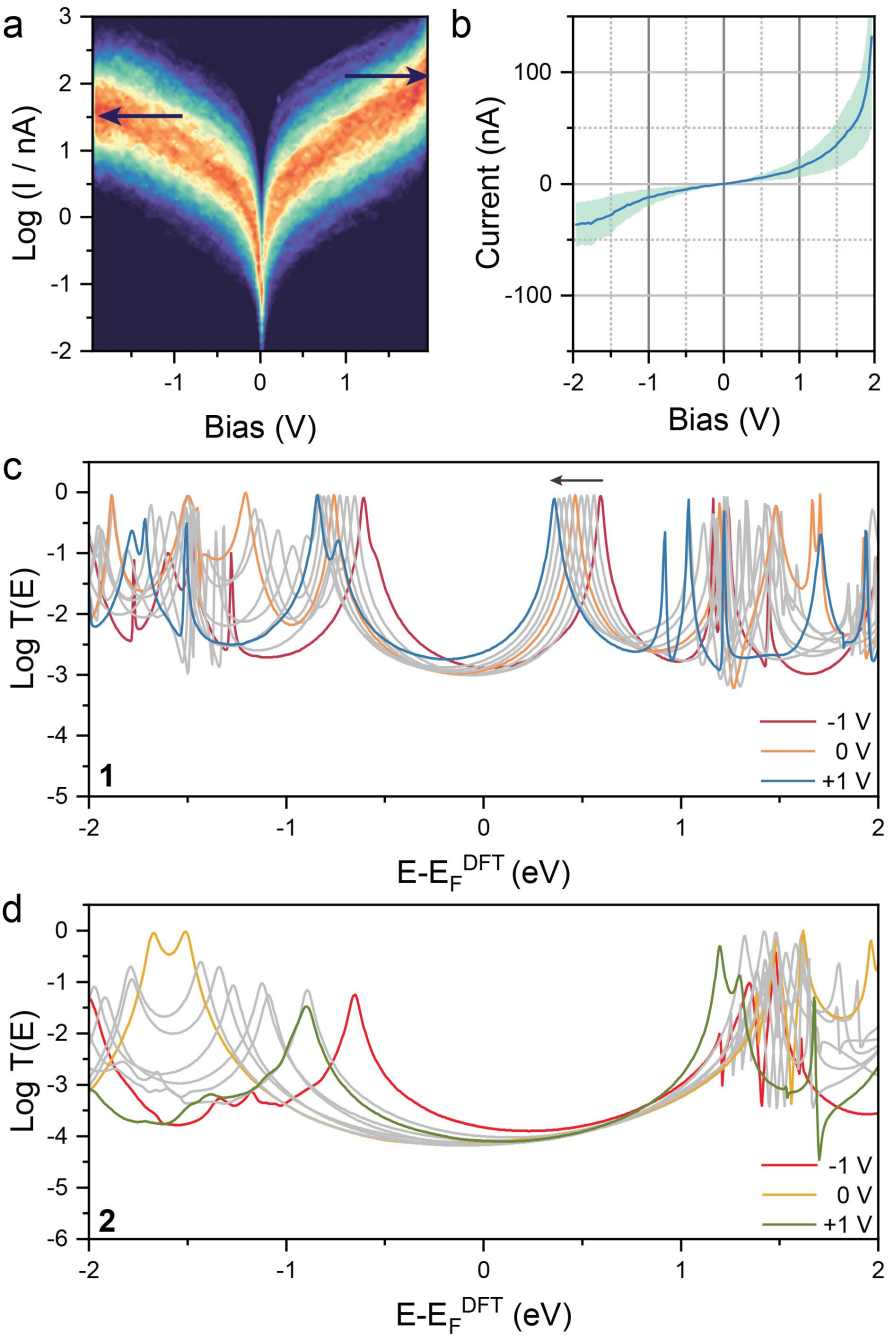
*I*–*V* behaviour of **1** and **2**. a) Semilogarithmic single‐molecule *I*–*V* heatmap for **1**. b) Gaussian fit of the data in (a), shown on a linear scale. Confidence interval is ±σ of the Gaussian fit. c) Bias‐dependent transmission coefficient calculations for **1**. d) Bias‐dependent transmission coefficient calculations for **2**. Data in (a) and (b) are acquired in mesitylene, with no electrochemical control and plots are compiled from >10 000 traces, using the algorithms described in the Supporting Information. The heatmap in (a) has been compiled with 20 bins per decade and 50 bins V^−1^. Intermediate, grey *T(E)* curves in (c) and (d) are obtained at 0.25 V increments (±0.75, ±0.5 and ±0.25 V).

Having established that charge transport through **1** is enhanced by the presence of a partially filled molecular orbital near the Fermi level, attention was turned to single‐molecule bias modulation experiments, with the aim of establishing the current‐voltage (*I*–*V*) behaviour of this compound. In these experiments, a modified STM‐BJ technique was used. The tip was driven into the substrate and then retracted in a staircase manner, with steps of size commensurate to the length of the molecule of interest (in this case, 1.1 nm). Between steps, the tip‐substrate bias was held at a stable value (+200 mV) for 25 ms, then stepped and ramped between +2 and −2 V at a rate of 80 V s^−1^ to obtain each *I*–*V* curve over a period of 50 ms; at the end of the ramp, the bias was stepped back to 200 mV and again held stable for further 25 ms. Data were then sliced and processed using automated algorithms described in the Supporting Information. The *I*–*V* characteristics of **1** are not linear, with the ohmic behaviour displayed between ±0.7 V giving way to clear evidence of current rectification above ±1.5 V (Figure 4a, b). At negative bias the current saturated at a value of ≈25 nA, while increasing rapidly to values >100 nA at positive bias, giving a rectification ratio *RR*_±2*V*_≅5. The tetrazine **2** showed no rectification or current saturation behaviour (see Supporting Information). It is worth noting that the *I*–*V* experiments performed here lack the coated tips and highly polar environment required to drive 2‐terminal (2‐electrode) electrochemistry[^44^, ^45^] and the redox state of **1** does not change during these measurements.

To better understand this phenomenon, we performed bias‐dependent non‐equilibrium Green's function calculations using the Gollum^[46]^ code, combined with the mean‐field Hamiltonian obtained from DFT. These bias‐dependent calculations demonstrate that the transmission spectrum is sensitive to the applied bias voltage, with the resonances shifting to lower energies when a positive voltage is applied, and conversely shifting to higher energies when negative bias is applied (see Supporting Information for more details). The transport resonance associated with the SUMO (or β‐LUMO) of **1** enters the bias window at positive bias voltages (leading to high currents, see arrow in Figure 4c) whereas it remains outside and does not contribute to charge transport at negative bias voltages, due to its shift to higher energies. We note that the bias dependence of the SOMO and SUMO resonances of **1** behave differently from those arising from the other (doubly (un)occupied) orbitals of **1** or the frontier orbitals of **2**, as the charge density distribution is different for different orbitals. For example, the charge density of the α‐SOMO is localised mainly on the verdazyl ring whereas that of the β‐SOMO is localised mainly on the aurophilic termini (Table S2 in the Supporting Information) and therefore the shift of resonances due to the applied bias for these orbitals does not follow the same trend. These phenomena combined lead to a distinct asymmetry of the *I*–*V* behaviour for the open‐shell **1**. Furthermore, the rectifying behaviour in our study is demonstrated by a structurally symmetrical molecule therefore arises within the framework of a coherent tunnelling mechanism, and is not due to the surrounding environment[^47^, ^48^] or different molecular interfaces to the source and drain electrodes,[^49^, ^50^, ^51^, ^52^] as in previously reported single‐molecule diodes. For the closed‐shell molecule **2**, where the Fermi energy is close to the middle of the HOMO–LUMO gap as is often the case in molecular junctions, the shift of transmission spectra by bias voltage does not lead to significant difference of the transmission coefficient near *E_F_* at biases of opposite polarity, and therefore there is no asymmetry in the *I*–*V* behaviour (Figure 4d).

## Conclusion

These studies have demonstrated that the 6‐oxoverdazyl radical **1** maintains its open‐shell character when incorporated into molecular junctions fabricated at room temperature. The open‐shell electronic character of **1** gives rise to a pair of transport resonances, arising from semi‐occupied (SOMO or α‐HOMO) and semi‐unoccupied (SUMO or β‐LUMO) molecular orbitals. The energy alignment of these resonances with the Fermi levels of the metal electrodes grants **1** significantly enhanced charge‐transport efficiency compared to its precursor **2**. The radical compound **1** can be reversibly electrochemically reduced to a closed‐shell anion within the junction, with the distinct charge‐transport properties of the two charge states giving rise to single‐molecule transistor behaviour. In addition, the bias‐dependent behaviour of the SUMO transport resonance imparts rectifying behaviour to junctions fabricated with **1**, with significantly greater current flowing under positive bias due to the migration of the transport resonances within the bias window. These results provide a strategy to incorporate radical compounds in single‐molecule devices, key to enabling technologies such as molecular spintronics and molecular thermoelectrics.

Supporting Information: The Supporting Information document contains details about the methods employed and additional single‐molecule conductance data and calculations.

## Author Contributions

PJL, SS and HS conceived the project. VJK and JZW synthesised the compounds used in this study and performed their characterisation. AV and RJN designed the charge transport experiments. SN performed the single‐molecule experiments and analysed the data with software written by AV. SS performed the computational studies. EG performed preliminary single‐molecule measurements. XQ trained SN in the electrochemical single‐molecule experiments and assisted data acquisition. MJ and NC performed cw‐EPR measurements. All authors contributed to the discussion of the results. AV, PJL and HS wrote the paper with contributions from all authors.

## Data Availability

Raw single‐molecule charge transport data, Labview Vis, and Python code used for its processing are available under a CC‐BY license in the University of Liverpool Data Catalogue at:

- DOI: 10.17638/datacat.liverpool.ac.uk/1529 (*STMBJ* and *EC‐STMBJ* measurements)
- DOI: 10.17638/datacat.liverpool.ac.uk/1513 (*I*–*V* measurements)

## Competing Financial Interests

The authors declare no competing financial interests.

## Conflict of interest

The authors declare no conflict of interest.

## Supporting information

As a service to our authors and readers, this journal provides supporting information supplied by the authors. Such materials are peer reviewed and may be re‐organized for online delivery, but are not copy‐edited or typeset. Technical support issues arising from supporting information (other than missing files) should be addressed to the authors.

Supporting Information
