# Supplementary material for: Redox‐Addressable Single‐Molecule Junctions Incorporating a Persistent Organic Radical
Source: Angew Chem Int Ed Engl. 2022 Apr 5;61(23):e202116985. doi: 10.1002/anie.202116985 (PMC9322687; doi:10.1002/anie.202116985)
Supplement: Supplementary file 1 — Supporting Information [file ANIE-61-0-s001.pdf]

Angewandte Chemie
Eine Zeitschrift der Gesellschaft Deutscher Chemiker

Supporting Information

# Redox-Addressable Single-Molecule Junctions Incorporating a Persistent Organic Radical

*S. Naghibi, S. Sangtarash, V. J. Kumar, J.-Z. Wu, M. M. Judd, X. Qiao, E. Gorenskaia, S. J. Higgins, N. Cox, R. J. Nichols*, H. Sadeghi*, P. J. Low*, A. Vezzoli**

# Table of Contents

1. Additional Experimental Details ........................................ 3

2. EPR Methods and Data ........................................ 4

2.1 Additional Details: ........................................ 5

3. Single-Molecule Conductance Measurements ........................................ 7

3.1 Additional STM-BJ Data ........................................ 8

4. Additional Electrochemical Data ........................................ 12

5. Computational Methods ........................................ 13

6. References ........................................ 16

# 1. Additional Experimental Details

Compounds **1** and **2** were prepared as described elsewhere.[1] Cyclic voltammetry measurements plotted in Figure 3 of the manuscript were conducted in a standard three-electrode cell, with Pt disc working electrode, Pt wire counter and Pt wire pseudo-reference electrodes, from solutions in 0.1 M $NBu_4PF_6$ / $CH_2Cl_2$, with data collected from an EmStat3+ potentiostat. The ferrocene/ ferrocenium ($E_{1/2}$ = 0 V) and decamethyl ferrocene/ decamethyl ferrocenium couples ($E_{1/2}$ = –0.55 V versus ferrocene / ferrocenium) were used as internal references for potential measurements.[2]

## 2. EPR Methods and Data

**Liquid solution EPR samples:** A solution of **1** was prepared in 100 µM concentration in dichloromethane ($CH_2Cl_2$), degassed by bubbling under Argon gas for 15 minutes. The sample was measured in a Q-band capillary (0.3 mm ID), placed inside an X-band quartz tube to minimize microwave dampening by $CHCl_3$.

**Frozen solution EPR samples:** A solution of **1** was prepared in 100 µM concentration 50/50 mix of $CHCl_3$/toluene. The sample was measured in a 3.8 mm OD X-band EPR tube at 120 K.

**Gold/substrate EPR samples:** Sample of **1** deposited onto Au coated glass substrate were also prepared for cw-EPR measurements. Substrates were incubated in 1 mM solution of **1** in chloroform for 48 hours, followed by rinsing and drying under flowed nitrogen gas. The gold-plated glass substrate was then inserted into a 3.8 mm OD X-band EPR tube.

**Continuous Wave cw-EPR:** measurements were performed at room temperature on a Bruker E500 spectrometer equipped with an ER4122 SHQ resonator. Spectra were recorded with a field modulation amplitude of 0.5 G and a power of 0.47 mW for the solution phase measurements, and a modulation amplitude of up 5 G and 4.7 mW for the gold/substrate measurements.

**EPR simulations:** Spectra were simulated using the EasySpin package[3] in MATLAB using the isotropic and fast-motion cw-EPR program *garlic* for the solution spectrum, and *pepper* for the solid state spectrum. Simulations were optimized using a Nelder-Mead minimization algorithm.

**EPR data:**The solution EPR spectrum of **1** in dichloromethane at room temperature is shown in Figure S1A. Its lineshape is typical of 6-oxo-verdazyls and its derivatives.[4,5] The nine-line structure comes about from the coupling of the unpaired electron spin to the four $^{14}N$ nuclei ($I$ = 1) of the central ring.[4] Owing to the symmetry of the molecule, the four nitrogens form two equivalent sets (Table S1). As shown in a previous publication by our group, the introduced phenyl rings at the 1- and 5-positions of the 6-oxo-verdazyl core has little effect on its electronic structure.[6] The frozen solution (solid state) EPR spectrum of **1** was also measured (Figure S1B). Owing to inhomogeneous line broadening and the molecules now no longer tumbling in solution, the EPR spectrum is characteristically less resolved, although some $^{14}N$ hyperfine structure is retained. Its g value (central crossing point) is slightly higher than in solution (2.005 *vs.* 2.0039).

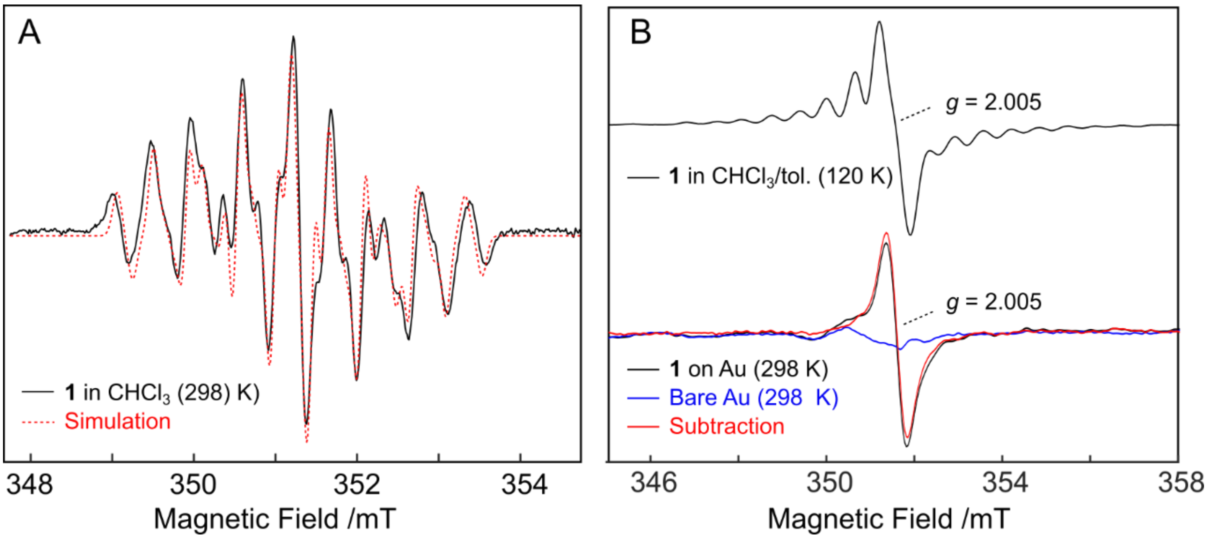


Figure S1: A) Room temperature CW-EPR (X-band ~9.4 GHz) of 0.1 mM **1** in $CHCl_3$ (black trace), and *EasySpin* simulation (red dotted trace). B) Top panel: CW-EPR (X-band ~9.4 GHz) frozen solution spectrum of **1** in $CHCl_3$/toluene measured at 120 K. Bottom panel: CW-EPR spectrum of **1** deposited onto a gold-on-glass substrate (black), compared to gold plated glass baseline (blue). The difference between the two spectra is shown in red.

We also measured the EPR spectrum of **1** deposited on a gold surface (Figure S1B). A radical signal is observed, which appears at the same g value of g~2.005 as the radical measured in frozen solution. The bare Au-coated substrate shows no large EPR signal in this spectral region, confirming the recorded signal arises from successful chemisorption and retention of **1** on the gold surface. The EPR spectrum of **1** on Au is of a similar width to the frozen solution spectrum, but does not clearly resolve any $^{14}$N hyperfine structure, suggesting the unpaired electron density across **1** may be more delocalized when attached to the gold surface. No significant orientation dependence of the gold plates was observed. At low temperature (~100 K) the radical signal was reduced significantly, supporting the assignment to a slow relaxing organic radical species.

Table S1: Fitting parameters for simulations of cw-EPR spectrum of **1,** shown in Fig. 2. Spectral features modelled using the EasySpin package *garlic.*[3] See text for atom assignment.

| g-value | Linewidth /mT | Hyperfine couplings /MHz | |
|---|---|---|---|
| | | a($N_{2,4}$) | a($N_{1,5}$) |
| 2.0039 | 0.14 | 18.0 | 12.5 |

## 2.1 Additional Details:

A basis set that describes the radical spin manifold can be built from the product of the eigenstates of the interacting electron ($S$ = 1/2) and nuclear ($^{14}$N, $I$ = 1) spins:

$$|S \quad M\rangle \otimes |I_1 \quad m_1\rangle \otimes |I_2 \quad m_2\rangle \otimes |I_3 \quad m_3\rangle \otimes |I_4 \quad m_4\rangle \qquad \text{Eq. S1}$$

Here, $S$ refers to the electronic spin state, $M$ refers to the electronic magnetic sublevel, $I_i$ refers to the nuclear spin state of $^{14}N$, and $m_i$ refers to the nuclear magnetic sublevels of each $^{14}N$. $S$ take the value of ½ and $M$ the values ±1/2. $I_i$ the value of 1 and $m_i$ the values -1, 0 and +1. The spin Hamiltonian that describes the spin manifold is:

$$\hat{H} = g\beta_e\vec{B}_0 \cdot \vec{S} + \sum_{i=1}^{4}\left(-g_n\beta_n\vec{B}_0 \cdot \vec{I}_i + a_i\vec{S} \cdot \vec{I}_i\right) \qquad \text{Eq. S2}$$

It contains (i) an electronic Zeeman term describing the unpaired electron's interaction with the applied magnetic field ($g$), (iv) a nuclear Zeeman term for each $^{14}N$ nucleus and the applied magnetic field, and (iii) an electron-nuclear hyperfine term ($a_i$) for each $^{14}N$ nucleus describing the magnetic interaction between the unpaired electron and each nucleus. Note that the nuclear quadruople term does not need to be considered when simulation the EPR spectrum.

Owing to the symmetry of the oxoverdazyl framework, the four $^{14}N$ hyperfine couplings represent two equivalent sets: two larger couplings $a_1$ and $a_2$; and two smaller couplings $a_3$ and $a_4$. In Table S1, $a_1$ and $a_2$ are described by the label $a(N_{2,4})$ and $a_3$ and $a_4$ are described by the label $a(N_{1,5})$ which makes use of the crystallographic labelling of the four nitrogen sites.

## 3. Single-Molecule Conductance Measurements

A modified Keysight 5500 STM was employed for the fabrication and characterisation of single-molecule junctions. STMBJ and EC-STMBJ (electrochemical control) experiments were performed using a custom 4-channel current amplifier based on the design by Meszaros et al.[7] The Keysight N9610A electronics controls the tip position and substrate bias, while the electrochemical potentials are applied by a bipotentiostat integrated in the STM controller. The 4-channel output of the preamplifier is recorded at 10 kSa/s by a National Instruments NI9215 USB DAQ with bespoke Python software. For single-molecule I-V measurements, the STM was equipped with a wide-bandwidth Femto DLPCA-200 single-channel preamplifier. A dual-channel Keysight 33522B Arbitrary Waveform Generator (AWG) imposes a bias to the substrate and controls the tip position, by applying a voltage ramp to the STM piezoelectric transducer through a Keysight N9447A breakout box. All signals (tip position, substrate bias and current amplifier output) are acquired simultaneously at 20 kSa/s with a National Instruments PXI system (24-bit PXI-4464 DAQ, PXIe-1062Q chassis, PXIe-PCIe8381 interface). A 10 kΩ resistor between the AWG and the substrate prevents overload of the preamp when in contact during the high-voltage ramps. Data acquisition is performed with bespoke Python code or Labview VIs. All experiments performed with Au tips cut from a spool of Au wire (Goodfellow, 99.99+%) and Au-on-mica substrates (Advent Research Materials Au 99.99+% evaporated on freshly cleaved Agar Scientific muscovite mica using a Korvus Technology Tau e-beam evaporator). STMBJ experiments and single-molecule I-V measurements performed in a 1 mM solution of the target molecular wire in mesitylene (TCI UK). EC-STMBJ experiments performed in a 1 mM solution of the target molecular wire in the ionic liquid 1-butyl-3-methylimidazolium triflate (IoLiTec), that was previously dehydrated by heating for >16 hours at 120 °C *in vacuo* (~8 mbar) in the presence of 20% w/w 4Å molecular sieves (Merck). For EC-STM experiments, the tip was coated with insulating wax (Apiezon W). A coiled platinum wire (Goodfellow 99.99+%) was used as counterelectrode and an electrochemically chloridised Ag wire (Goodfellow 99.999+%) was used as reference electrode.[8] In all experiments the tip is held at ground.

For the determination of molecular conductance using regular *STMBJ* methods, all acquired data was used without further selection. For the measurements of the $I - V$ characteristics, we used a method described in details elsewhere,[9] with data collected from junction formed with bare-wire tips to avoid two-electrode electrochemistry In brief, data is acquired using a staircase ramp applied to the piezo transducer, with abrupt stretches of 1.1 nm followed by a 100 ms "hold" portion. During the hold, the bias is held at a constant value of 200 mV for 25 ms, and then ramped between 2 and -2 V at a rate of 80 V/s. After the ramp, the bias is held again at a constant value of 200 mV until the end of the hold. An automated algorithm was then used to analyse the data. First, the algorithm sliced the traces between abrupt stretches, by calculating the second derivative of the piezo signal and cutting when its value went above a threshold. After that, the average conductance of the junction during the fixed bias sections is calculated, and the algorithm only selects traces where this value falls within one standard deviation from the most probable molecular conductance, as determined by

regular *break-junction* measurements. This process filters out data where no junctions was formed, or where the molecular junction did not survive the whole bias modulation process. On average, 30-40% of traces are retained by our algorithm. The resulting slices, now only relative to stable junctions are used without further processing and compiled into 2d heatmaps as shown in the manuscript and later in this document.

## 3.1 Additional STM-BJ Data

In addition to the data presented in the manuscript, additional data and details are provided here. 2D maps and full STMBJ histograms are presented below, for experiments performed in mesitylene.

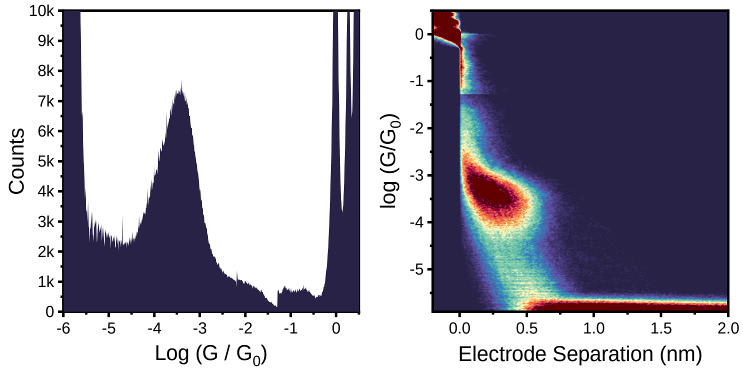


Figure S2: STM-BJ measurements on **1**. 0.2V bias, 1 mM in mesitylene. Histogram and 2D density map compiled from 7807 individual scans, no data selection, 100 bins / decade, 100 bins / nm.

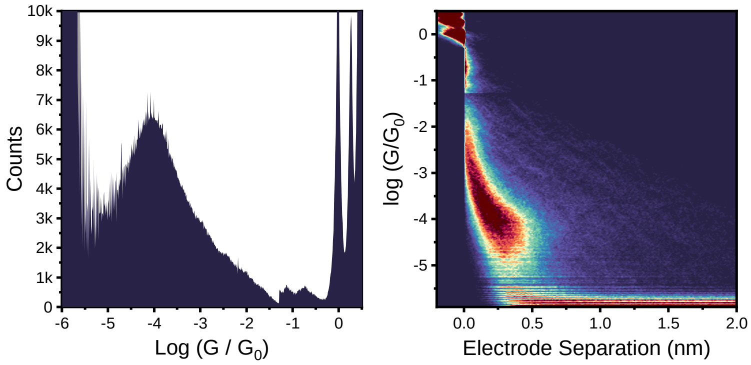


Figure S3: STM-BJ measurements on **2**. 0.2V bias, 1 mM in mesitylene. Histogram and 2D density map compiled from 7322 individual scans, no data selection, 100 bins / decade, 100 bins / nm.

Compounds **1** and **2** were also measured under atmospheric conditions, on a sub-monolayer on Au pre-adsorbed from a 1 mM solution of the target molecule in acetone. Results are comparable to those obtained in mesitylene, with the verdazyl **1** showing higher charge transport efficiency than its non-radical precursor **2**.

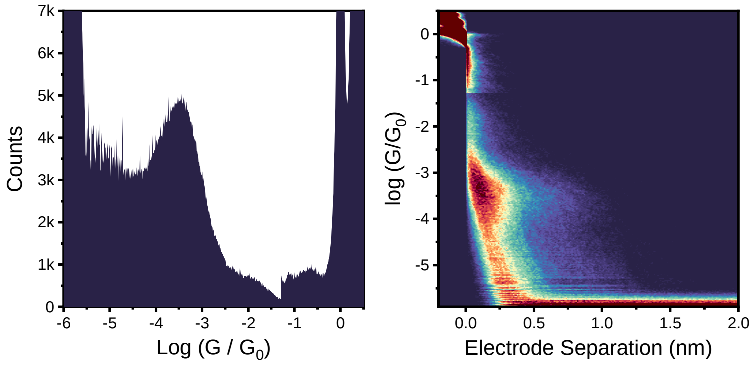


Figure S4: STM-BJ measurements on **1**. 0.2V bias, pre-adsorbed from a 1 mM acetone solution and measured under atmospheric conditions. Histogram and 2D density map compiled from 6672 individual scans, no data selection, 100 bins / decade, 100 bins / nm.

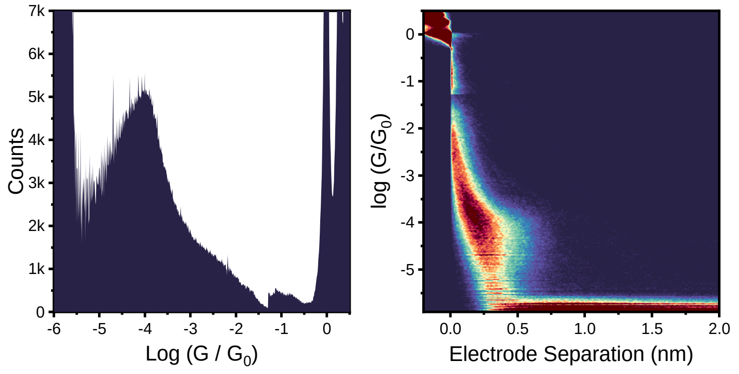


Figure S5: STM-BJ measurements on 2. 0.2V bias, pre-adsorbed from a 1 mM acetone solution and measured under atmospheric conditions. Histogram and 2D density map compiled from 7213 individual scans, no data selection, 100 bins / decade, 100 bins / nm.

Single-molecule electrochemical break-junction experiments were also performed over a wider potential range than shown in the main manuscript, exploring an electrochemical window 1.8 V wide. Data at potentials > 0.5 V vs $Fc/Fc^{+}$ showed greatly increased instrumental noise and no apparent junction formation. From the cyclic voltammetry shown in the main paper we expect **1** to be oxidised at these potentials, but the technical difficulties detailed earlier prevented us from characterising the charge transport properties in the +1 state. It is indeed possible that **1** in its cationic state is not soluble in the ionic liquid we employed, and therefore precipitates out of solution and/or deposits on the electrode surface. A wide-range conductance vs potential map is presented in Figure S6. It should be noted here that no data could be acquired at potentials > 0.5 V

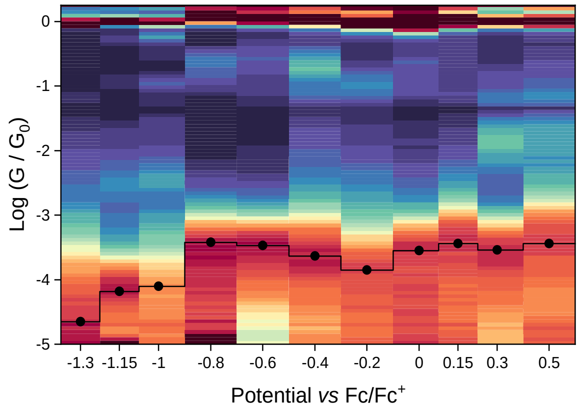


Figure S6: Single-molecule conductance data for **1** across the full electrochemical window explored. 0.2 V bias, 1 mM in 1-butyl-methylimidazolium triflate, Pt counterelectrode, Ag/AgCl reference electrode.

In addition to the full electrochemical map, we present here data collected after cycling **1** between the radical and the anionic state. Upon return to a small negative potential, the high conductance signal could be recovered.

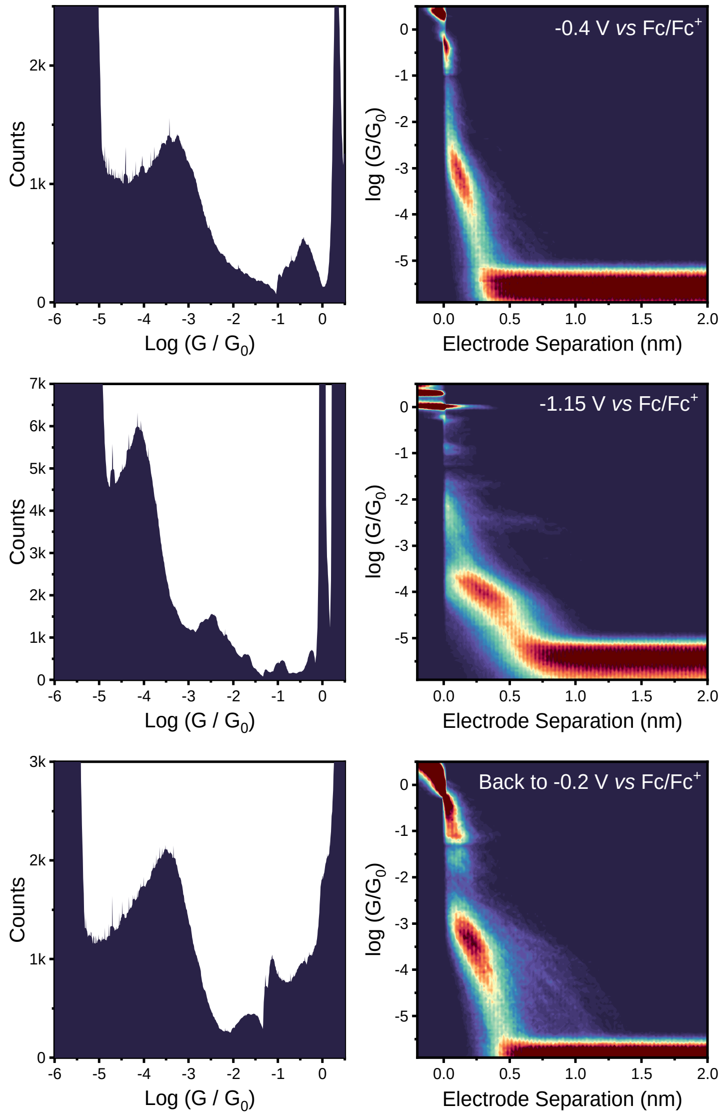


Figure S7: STM-BJ measurements on **1**. 0.2V bias, 1 mM in 1-butyl-methylimidazolium triflate, Pt counterelectrode, Ag/AgCl reference electrode. Measurements were performed at 0.4 V potential *vs* $Fc/Fc^+$ (open circuit), then the potential was decreased to -1.15 V *vs* $Fc/Fc^+$, and then returned to -0.2 V *vs* $Fc/Fc^+$. Data compiled from, respectively top to bottom, 3258, 5037, and 3160 individual scans. Histograms and 2d density maps compiled with no data selection, 100 bins / decade, 100 bins / nm.

Single-Molecule I-V data for **2** and a comparison with **1** are also provided below.

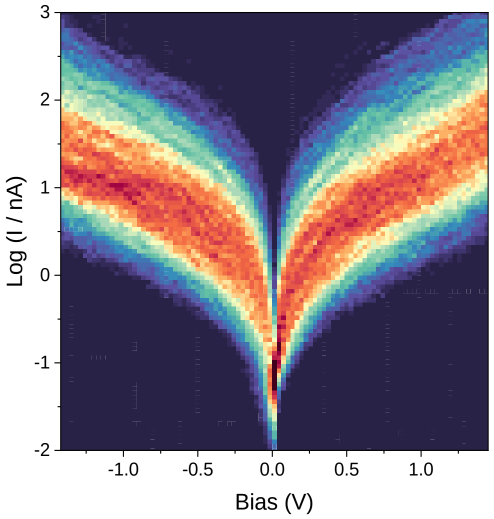


Figure S8: Semilogarithmic single-molecule I-V heatmap for **2**. Heatmap compiled with data from >10000 traces, using the algorithms described earlier, with 20 bins per current decade and 50 bins per Volt.

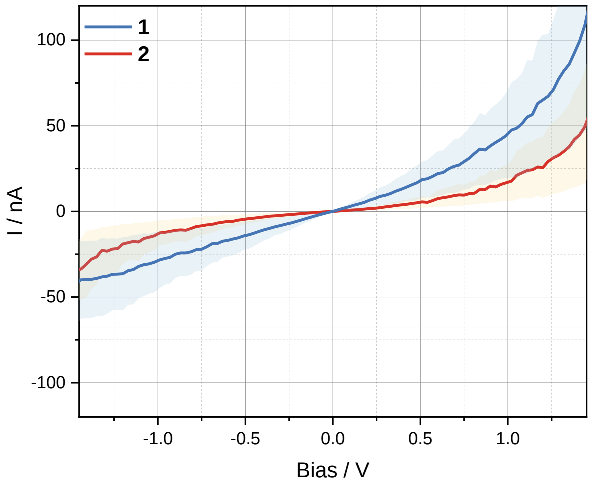


Figure S 9: Comparison between Gaussian fits to single-molecule I-V heatmaps for **1** and **2**. Confidence interval is ±σ of the Gaussian fit. The open-shell **1** displayed remarkable asymmetry in its I-V behaviour, while the close-shell **2** has almost perfectly symmetric I-V response ($RR_{\pm 1.5V} \cong 1.5$).

# 4. Additional Electrochemical Data

The electrochemical behaviour of **1** is shown in the main paper on a Pt electrode, in $CH_2Cl_2$ with tetrabutylammonium hexafluorophosphate as support electrolyte. We also recorded CVs of **1** in the environment used for the STMBJ measurements, using an Au working electrode, a Pt counter-electrode, and a Pt pseudo-reference electrode in 1-butyl-3-methylimidazolium triflate.

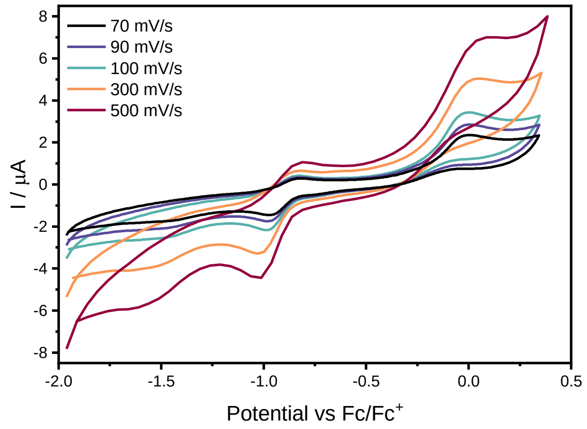


Figure S10: Cyclic voltammetry of **1** in the ionic liquid 1-butyl-3-methylimidazolium triflate. Au disk working electrode, Pt wire counter-electrode and Pt wire pseudo-reference electrode. Potential referenced to the ferrocene/ferrocenium redox couple using ferrocene as internal standard.

As can be observed in Figure S10, the reduction of **1** retains good chemical reversibility and quasi-reversible electrochemical behaviour. **1** can be cycled between the neutral radical and the anionic state as shown by the wave in the CV centred at around -900 mV (vs. $Fc/Fc^+$), with a peak-to-peak separation of 140 mV at a scan speed of 70 mV/s. The oxidation of **1** to the cationic state shows a peak at about 0 V at lower sweep rates, which is, however, completely irreversible. As discussed in the main text, we attribute this to the insolubility of the cationic species in 1-butyl-3-methylimidazolium triflate, leading to precipitation and preventing its reduction as the voltage is made less positive.

# 5. Computational Methods

**Characterisation of gas phase molecules:** The optimized geometry of **1** and **2**, their molecular orbitals and spin density of **1** are calculated density functional theory. We perform these calculations with two different codes. SIESTA (using parameters described below) and Gaussian v16 (with B3LYP hybrid functional, qzvp basis set and tight convergence criteria) and obtained similar results.

**Molecules between gold electrodes:** The optimized geometry and ground state Hamiltonian and overlap matrix elements of each structure studied in this paper was self-consistently obtained using the SIESTA implementation[10] of density functional theory (DFT). SIESTA employs norm-conserving pseudo-potentials to account for the core electrons and linear combinations of atomic orbitals to construct the valence states. The local density approximation (LDA) of the exchange and correlation functional is used with the CA parameterization a double-ζ polarized (DZP) basis set, a real-space grid defined with an equivalent energy cut-off of 150 Ry. The geometry optimization for each structure is performed to the forces smaller than 20 meV/Å.

**Quantum transport calculations:** The mean-field Hamiltonian obtained from the converged DFT calculation was combined with GOLLUM[11,12] implementation of the non-equilibrium Green's function method[12] to calculate the phase-coherent, elastic scattering properties of the each system consist of left gold (source) and right gold (drain) leads and the scattering region. The transmission coefficient *T(E)* for electrons of energy *E* (passing from the source to the drain) is calculated via the relation: $T(E) = Trace(\Gamma_R(E) G^R(E) \Gamma_L(E) G^{R\dagger}(E))$. In this expression, $\Gamma_{L,R}(E) = i\left(\sum_{L,R}(E) - {\sum_{L,R}}^{\dagger}(E)\right)$ describe the level broadening due to the coupling between left (L) and right (R) electrodes and the central scattering region, $\sum_{L,R}(E)$ are the retarded self-energies associated with this coupling and $G^R = (ES - H - \sum_L - \sum_R)^{-1}$ is the retarded Green's function. The Transport properties is then calculated using the Landauer formula $G(E_F, T) = G_0 \int_{-\infty}^{+\infty} dE\, T(E)(-\partial f(E, T, E_F)/\partial E)$, where $f = (e^{(E-E_F)/k_B T} + 1)^{-1}$ is the Fermi-Dirac probability distribution function, *T* is the temperature, $E_F$ is the Fermi energy, $G_0 = 2e^2/h$ is the conductance quantum, *e* is electron charge and *h* is the Planck's constant.

For non-equilibrium current voltage calculations, we employ non-equilibrium Green's function method implemented in Gollum. First we calculate bias dependent transmission coefficient *T*(*E*,*V*) and calculate room temperature current for each bias voltage using:

$$I(V) = \frac{e}{h} \int_{-\infty}^{+\infty} dE\, T(E, V)(f(E, -eV/2) - f(E, eV/2))$$

We note that the actual electrode structure is unknown in the experiment, therefore it is not clear that how much of potential drops happens across the junction, at the electrode and its surface and at the interface between molecule and

electrodes. Therefore, we can only compare qualitatively our bias dependent calculations with the experimental results. That is why we choose a ±1 V bias window and demonstrate that the resonances due to frontier orbitals are shifted by the bias voltage. The resonances are closer to DFT Fermi energy in the radical compound **1** and therefore, their small bias-induced shift has a large effect on the transported current, in contrast to the closed shell molecule **2** where resonances are far away from DFT Fermi energy and their shift do not affect current significantly.

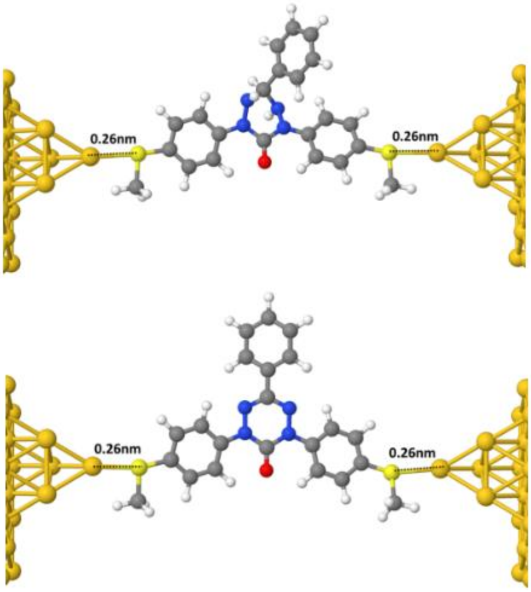


Figure S11: Structure of **1** (bottom) and **2** (top) between two gold electrodes in a single-molecule junction configuration.

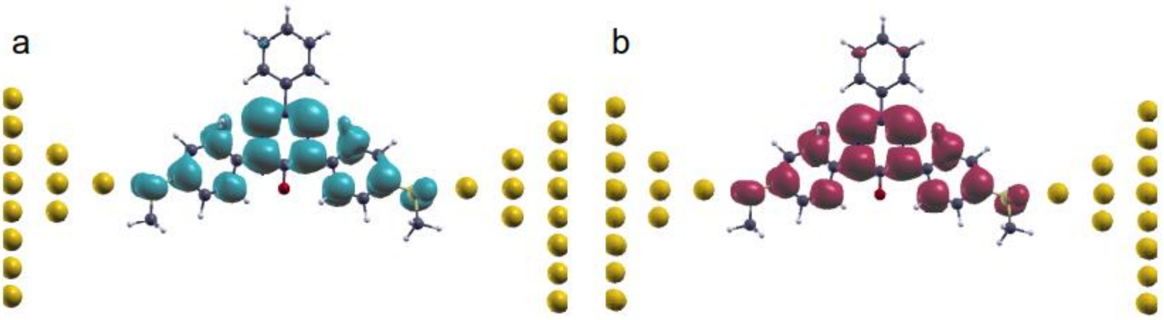


Figure S12: Local density of state for energy range ±0.1 eV centred around the maximum of SOMO (a) and SUMO (b) transmission resonances shown in Figure 4b of the main paper for V = 0 V.

Table S2: Molecular orbitals for **1** (left) and **2** (right).

| **Frontier Orbitals for 1** | **Frontier Orbitals for 2** |
|---|---|
| -1.66 (eV) 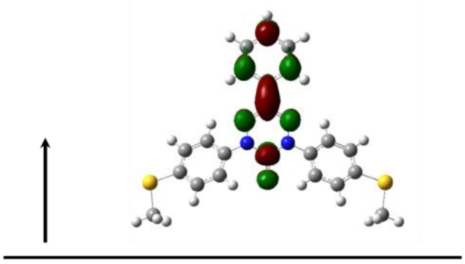 α-SUMO | -1.24 (eV) 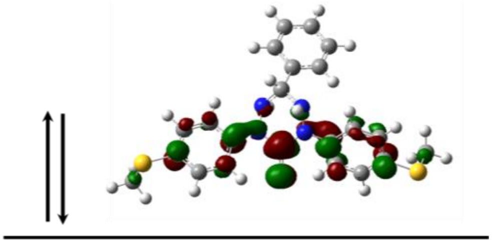 LUMO |
| -3.24 (eV) 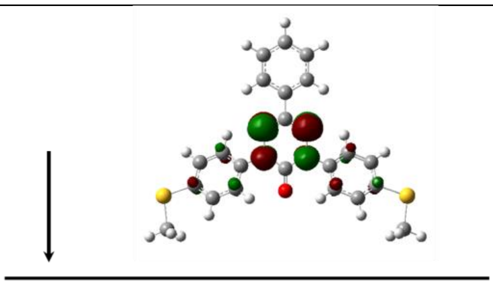 β-SUMO | |
| -5.46 (eV) 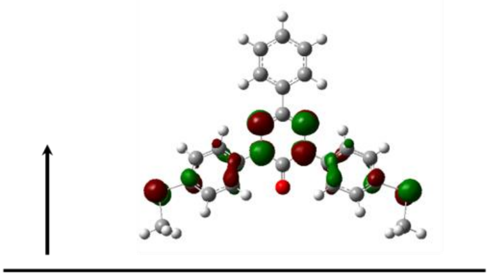 α-SOMO | |
| -6.02 (eV) 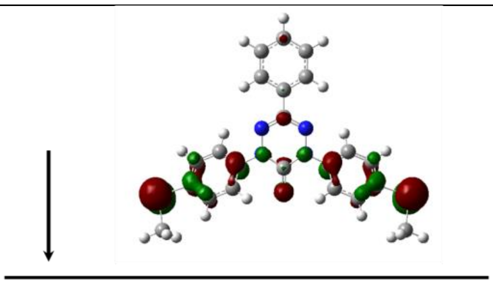 β-SOMO | -6.25 (eV) 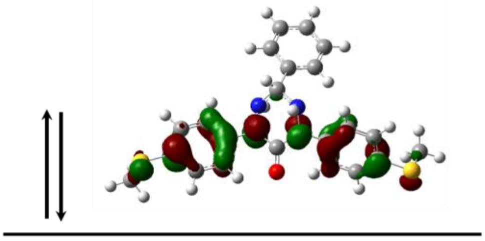 HOMO |

# 6. References


[1] V. J. Kumar, J.-Z. Wu, M. Judd, E. Rousset, M. Korb, S. A. Moggach, N. Cox, P. J. Low, *J. Mater. Chem. C* **2022**, *10*, 1896–1915.

[2] J. B. G. Gluyas, A. J. Boden, S. G. Eaves, H. Yu, P. J. Low, *Dalt. Trans.* **2014**, *43*, 6291–6294.

[3] S. Stoll, A. Schweiger, *J. Magn. Reson.* **2006**, *178*, 42–55.

[4] C. L. Barr, P. A. Chase, R. G. Hicks, M. T. Lemaire, C. L. Stevens, *J. Org. Chem.* **1999**, *64*, 8893–8897.

[5] E. C. Paré, D. J. R. Brook, A. Brieger, M. Badik, M. Schinke, *Org. Biomol. Chem.* **2005**, *3*, 4258.

[6] R. O. Fuller, M. R. Taylor, M. Duggin, A. C. Bissember, A. J. Canty, M. M. Judd, N. Cox, S. A. Moggach, G. F. Turner, *Org. Biomol. Chem.* **2021**, *19*, 10120–10138.

[7] G. Mészáros, C. Li, I. Pobelov, T. Wandlowski, *Nanotechnology* **2007**, *18*, 424004.

[8] A. Saheb, J. Janata, M. Josowicz, *Electroanalysis* **2006**, *18*, 405–409.

[9] C. Wu, X. Qiao, C. M. Robertson, S. J. Higgins, C. Cai, R. J. Nichols, A. Vezzoli, *Angew. Chem. Int. Ed.* **2020**, *59*, 12029–12034.

[10] J. M. Soler, E. Artacho, J. D. Gale, A. García, J. Junquera, P. Ordejón, D. Sánchez-Portal, *J. Phys. Condens. Matter* **2002**, *14*, 2745–2779.

[11] J. Ferrer, C. J. Lambert, V. M. García-Suárez, D. Z. Manrique, D. Visontai, L. Oroszlany, R. Rodríguez-Ferradás, I. Grace, S. W. D. Bailey, K. Gillemot, et al., *New J. Phys.* **2014**, *16*, 093029.

[12] H. Sadeghi, *Nanotechnology* **2018**, *29*, 373001.
